# To Eat or Not to eat: A Review of the Relationship between Chocolate and Migraines

**DOI:** 10.3390/nu12030608

**Published:** 2020-02-26

**Authors:** Magdalena Nowaczewska, Michał Wiciński, Wojciech Kaźmierczak, Henryk Kaźmierczak

**Affiliations:** 1 Department of Otolaryngology, Head and Neck Surgery, and Laryngological Oncology, Faculty of Medicine, Ludwik Rydygier Collegium Medicum in Bydgoszcz, Nicolaus Copernicus University, M. Curie 9, 85-090 Bydgoszcz, Poland; 2 Department of Pharmacology and Therapeutics, Faculty of Medicine, Collegium Medicum in Bydgoszcz, Nicolaus Copernicus University, M. Curie 9, 85-090 Bydgoszcz, Poland; 3 Department of Sensory Organs Examination, Faculty of Health Sciences, Collegium Medicum in Bydgoszcz, Nicolaus Copernicus University, M. Curie 9, 85-090 Bydgoszcz, Poland

**Keywords:** chocolate, cocoa, headache, migraine, trigger factor, polyphenols, flavonoid, flavonol, theobromine, magnesium

## Abstract

Migraine is a chronic disorder with episodic attacks, and patients with a migraine often report that certain factors can trigger their headache, with chocolate being the most popular type of food-based trigger. Many studies have suggested a link between chocolate and headaches; however, the underlying physiological mechanisms are unclear. As premonitory symptoms may herald migraine attacks, a question arises regarding whether eating chocolate before a headache is a consequence of a food craving or indeed a real trigger. Here, we aim to summarize the available evidence on the relationship between chocolate and migraines. All articles concerning this topic published up to January 2020 were retrieved by searching clinical databases, including EMBASE, MEDLINE, PubMed, and Google Scholar. All types of studies have been included. Here, we identify 25 studies investigating the prevalence of chocolate as a trigger factor in migraineurs. Three provocative studies have also evaluated if chocolate can trigger migraine attacks, comparing it to a placebo. Among them, in 23 studies, chocolate was found to be a migraine trigger in a small percentage of participants (ranging from 1.3 to 33), while all provocative studies have failed to find significant differences between migraine attacks induced by eating chocolate and a placebo. Overall, based on our review of the current literature, there is insufficient evidence that chocolate is a migraine trigger; thus, doctors should not make implicit recommendations to migraine patients to avoid it.

## 1. Introduction

Headaches have emerged as a great public health concern, with almost three billion people estimated to suffer from them worldwide, and, among them, 1.04 billion people suffer from migraines [1]. A migraine is a type of primary headache with recurrent attacks, typically with unilateral, pulsating, severe headaches that last from 4 to 72 h, with accompanying nausea, photophobia, phonophobia, and sometimes even transient neurological symptoms [2]. Individuals with migraines commonly report that certain factors can trigger a migraine attack [3,4,5,6,7]. Trigger factors are defined as measurable endogenous or exogenous exposures which increase the probability of an attack over a short period of time [8]. The most frequent migraine precipitating factors are stress, fatigue, fasting, lack of sleep, and weather. Foods are reported as a migraine trigger by approximately 20% of individuals with migraines, and chocolate is thought to be one of the most popular food-based triggers of a migraine [9]. Migraine sufferers are often recommended to avoid triggers, which can lead to restrictive changes in lifestyle, with further unhappiness and frustration [8,10]. However, the evidence to support these recommendations is insufficient [10,11]. The aim of this review is to examine the relationship between chocolate and migraines, and to check whether chocolate avoidance may be of benefit to certain patients.

### 1.1. Chocolate Composition

Chocolate consists of cocoa powder, cocoa butter, sugar, and milk powder (in the case of milk chocolate). The two first ingredients are found naturally in the cocoa bean, and the combination of both makes cocoa mass (also known as cocoa liquor) [12]. Cocoa, the raw material for chocolate, is a dry, powdered product made from the beans of the *Theobroma cacao* plant. It contains various polyphenols (more than most foods, even tea and red wine), particularly flavonoids, with a subclass called flavanols (epicatechin and oligomeric procyanidins), which are biologically active and may thus affect human health [13,14]. The beneficial effect of polyphenols on health is thought to be associated with its high content of antioxidants. Other active components of cocoa are methylxanthines (caffeine and theobromine, with a 1:5 ratio), as well as serotonin, its precursor, tryptophan, and β-phenylethylamine (PGA) [15,16,17]. Moreover, cocoa is a significant source of many vitamins and minerals important in the human diet, mostly magnesium, zinc, selenium, copper, potassium, riboflavin, and iron [18,19]. We distinguish dark chocolate, milk chocolate, and white chocolate [19]. It is worth noting that the highest concentration of flavonoids and minerals is found in dark chocolate, particularly in 90% cocoa-containing chocolate [13,18,20]. That is why dark chocolate is preferred over milk and white chocolate [14]. It should also be emphasized that cocoa is a rich source of fibre (26%–40%), proteins (15%–20%), carbohydrates (about 15%), and lipids (10%–24%) [19,21].

### 1.2. Chocolate and Health

A great amount of data suggests that cocoa has several important biological effects, mostly antioxidant, cardiovascular, anti-inflammatory, and metabolic effects [14]. It should be emphasized that most of the health benefits attributable to chocolate are associated with consuming the dark chocolate [19]. The available data indicate that chocolate consumption is connected with a reduced risk of acute myocardial infarction, death via cardiovascular disease, diabetes, and stroke; however, the evidence is weak [22]. Some research suggests a dose-response connection between chocolate consumption and the risk of all-cause mortality, heart failure, coronary heart disease, type 2 diabetes, hypertension, and colorectal cancer [23]. Another review shows that moderate consumption of chocolate is beneficial for a variety of conditions including hypertension, coronary heart disease, cholesterol, cerebrovascular accidents, heart failure, and peripheral vascular disease [24]. The cardioprotective effect of chocolate is probably associated with nitric oxide (NO). Flavanols stimulate endothelial NO synthase (eNOS) activity, leading to increased NO generation. This increase may be responsible for vasodilation and blood pressure reduction [13]. Another effect of NO enhancement is the prevention of leukocyte adhesion and migration, as well as smooth muscle cell proliferation, platelet adhesion, and aggregation [25].

A number of human and animal studies have documented the beneficial effects of polyphenols on the central nervous system (CNS), which is possibly connected to their anti-inflammatory activity [20]. Studies have demonstrated that catechin and epicatechin are able to cross the blood–brain barrier (BBB), with greater efficacy for epicatechin [26]. Both, as well as other flavonoids, can accumulate in the brain, and their action on the central nervous system is potentially sanogenetic for cognition, vision, and neuroprotection [27]. Traditionally, products containing xanthines, such as those derived from tea leaves, coffee, or cocoa beans, are known to stimulate the functioning of nervous systems. Another mechanism is associated with NO generation, with further vasodilatation and increased cerebral blood flow (CBF) and blood perfusion, not only in the CNS, but also in the peripheral nervous system. This CBF increase enhances the oxygen and glucose supply to neurons, helping to get rid of waste metabolites in the brain and sensory organs, but also stimulating angiogenesis in the hippocampus [13,20]. Increased blood flow in the middle cerebral artery may also account for protective effects in the course of stroke. Moreover, evidence exists that cocoa flavanols have beneficial effects on the progression of Parkinson’s and Alzheimer’s disease [20]. Chocolate also contains PGA, which is produced during the thermal processing of cocoa [17]. This neurotransmitter may be associated with specific psychological disorders, like attention deficit hyperactivity disorder, depression, and schizophrenia [17]. Cocoa polyphenols not only behave as powerful free-radical scavengers, but also modulate the microbial population of the human gut. It has been demonstrated that a diet containing 10% cocoa is able to modify the intestinal immune status as well as the composition of microbiota [28]. Moreover, evidence exists of the antinociceptive effects of polyphenolic compounds, particularly in animal models, where neuropathic, inflammatory, and nociceptive pains are reduced and alleviated by polyphenols [29]. Similarly, Costa de Miranda et al. demonstrated that the intake of polyphenol-rich foods is associated with a lower number of tender points and a better quality of life for people with fibromyalgia [30]. Another study has revealed, in a laboratory setting, that individuals who habitually consume greater amounts of caffeine as part of their daily diet demonstrate diminished sensitivity to painful stimuli [31]. As caffeine is naturally found in cocoa beans, it may also be one of the mechanisms of chocolate and pain association. In a very interesting study, Bastian et al. showed that participants enjoyed chocolate more after an experience of pain compared to completing a similar but non-painful task, meaning that physical pain increases the pleasantness derived from chocolate consumption [32].

Although much evidence about the health benefits of chocolate exists, one should remember that it is a high calorie food, and, if overused, may lead to weight gain and obesity, especially when more than 30 g/day is consumed [13,21,25,33]. One-hundred grams of cocoa, dark chocolate, and milk chocolate contains 355, 515, and 545 kcal, respectively [19]. However, a recent review has shown that cocoa or dark chocolate supplementation for at least 4 weeks at 30 g/day has a favorable effect on body mass index (BMI) and weight [33].

### 1.3. Chocolate and Migraines as a Potential Mechanism of Action

Migraine patients frequently describe that certain foods may induce or even exaggerate the severity of a headache attack [3,4,5,7,34,35,36,37]. Chocolate is the most popular food trigger of a migraine, and it has been epidemiologically implicated in triggering migraine attacks, and the classic advice given by doctors to patients with migraines is to avoid it [11]. Martin et al., in a review of the role of diet in the prevention of headaches, found that chocolate has been self-reported to be a precipitant for migraine headaches in 2%–22% of migraine sufferers [38]. Possible geographic variations in this matter may exist, as none of the patients among the Japanese and India survey groups reported chocolate consumption as a potential trigger of migraines [39,40].

The question arises: Why does chocolate induce a migraine attack? One possible explanation is that flavanols stimulate eNOS activity, leading to increased NO generation, which may lead to vasodilatation [13]. However, the role of vasodilation in migraines is unclear, and recent findings challenge its necessity [41]. There are also some ambiguities regarding the influence of chocolate on NO production. On the one hand, a great number of studies indicate that cocoa flavanols increase NO levels, while other studies have surprisingly found that cocoa reduces NO production by inhibiting the expression of NO synthase [20,42,43,44]. Another possible link between chocolate and migraine attack is serotonin. The concentrations of this neurotransmitter increase during a migraine attack. Cocoa is also suggested to play a role in the release of serotonin, which is proposed to be involved in the pathogenesis of migraines. Serotonin and its precursor tryptophan were found in chocolate, with the highest serotonin level in chocolate found with a cocoa content of 85% [15]. Theoretically, it is possible that by increasing the serotonin level, chocolate consumption may induce a migraine attack. Nevertheless, existing studies have not confirmed this theory [45]. Phenylethylamine is also reported as a neurotransmitter that can initiate migraine-type headaches in susceptible individuals. In an animal study, PGA produced significant increases in CBF and cerebral oxygen consumption during the first 40 min of infusion. In contrast, a further increased phenylethylamine concentration constricted the cerebral bed (cerebral blood flow reduced by 28%), producing a pattern of vascular events similar to those seen in migraines [46].

Although there is a lack of data showing the beneficial effect of chocolate on migraines, theoretically such a link is possible. First of all, chocolate contains many vitamins and minerals, among them magnesium and riboflavin, which are recommended in migraine prevention [47]. Magnesium plays an important role in neuromuscular conduction and nerve transmission and also acts as a protective agent against excessive excitation, which causes neuronal cell death. There is strong evidence regarding the beneficial effect of magnesium for chronic pain conditions and migraines [48,49]. One should remember that chocolate is a great source of Mg (with up to 252.2 mg of Mg per 100 g of chocolate). Another vitamin found in chocolate, riboflavin, was also reported to be efficient in the reduction of the frequency of migraines in adult patients [50]. It is known that migraine sufferers have chronically decreased serotonin levels, while the concentration increases during an attack [51]. Tryptophan is an essential serotonin precursor; thus, its depletion, which can occur with decreased dietary tryptophan intake, may increase the susceptibility to migraine-associated symptoms. One study showed that subjects who had a greater intake of tryptophan per day had reduced odds of developing migraine by approximately 54%–60% [45]. This means that chocolate, which contains both tryptophan and serotonin, by increasing serotonin levels, may also decrease the migraine frequency. Another study has shown that a cocoa-enriched diet suppresses stimulated levels of the inducible form of nitric oxide synthase (iNOS) [43]. NO is an important biological regulator and key mediator in migraines, where it regulates neurotransmission and vasodilation. NO levels increase in jugular venous plasma during a migraine attack, and additionally, iNOS inhibitors are effective in migraine treatment [52]. Therefore, it is possible that chocolate contains biologically active compounds that may reduce the incidence of migraine attacks by inhibiting NO synthase production.

CGRP (calcitonin gene-related peptide) is a neuropeptide that is released from the peripheral nerve, central nerve endings, and within the trigeminal ganglion. The release of CGRP starts a cascade of events, including the increased synthesis of nitric oxide, vasodilation, and causing mast cells to release inflammatory mediators and the sensitization of the trigeminal nerves, thus playing an essential role in the pathophysiology of migraines. The concentration of CGRP is elevated in the plasma of people with episodic and chronic migraines between and during migraine attacks; moreover, the administration of CGRP elicits a headache and sometimes a delayed migraine in migraineurs [53,54]. It should be emphasized that CGRP receptor antagonists are effective in migraine treatment. Cady et al. demonstrated that a cocoa-enriched diet prevents inflammatory responses in trigeminal ganglion neurons by inhibiting the expression of CGRP [43]. Abbey et al. showed that *Theobroma cacao* extract can repress the stimulated CGRP release by a mechanism that likely involves the blockage of calcium channel activity [42].

Depression and other mood diseases often coexist with migraines and can exaggerate their course. Chocolate is known to have mood-enhancing properties, mostly due to its orosensory properties, psychoactive ingredients, and the activation of neural reward pathways [55]. The consumption of chocolate may be associated with an improvement in the mood state, the attenuation of negative moods, or reduced odds of clinically relevant depressive symptoms [55,56].

Another mechanism by which chocolate may have a beneficial effect on migraines is the influence on the microbial population of the human gut [19]. Martami et al. demonstrated that a probiotic mixture could be an effective and beneficial supplement to treat migraine headaches in both chronic and episodic migraine sufferers [57]. As cocoa polyphenols may modulate the microbial population of the human gut, they may be another mechanism for treating migraines.

**Figure 1:**
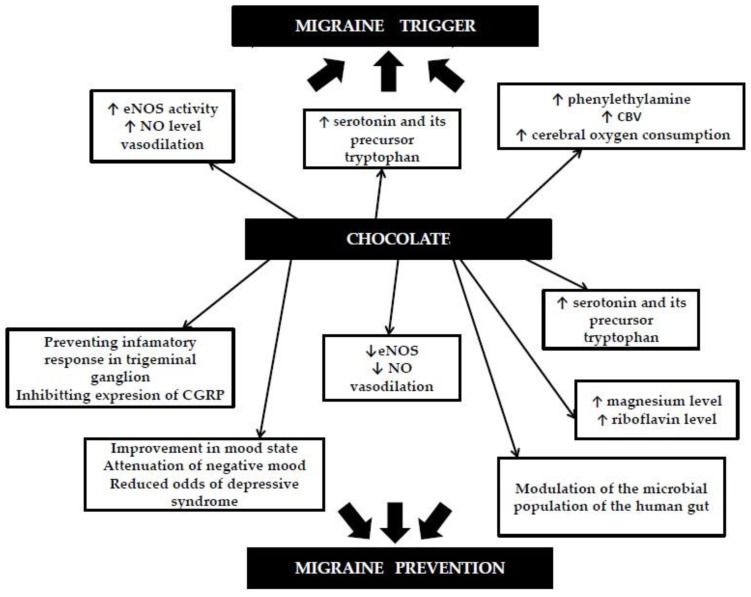
Possible mechanisms by which chocolate may trigger or prevent migraine attacks (based on our literature review).

All of this evidence shows that chocolate may act not only as a migraine trigger, but also as a protective factor, possibly decreasing the probability of an attack over a period of time (Figure 1).

### 1.4. Migraine Triggers

Trigger factors are events or exposures (endogenous or exogenous) that are linked to an increased probability of an attack over a relatively brief period of time [8]. According to a review based on 25 publications, the “top 10” migraine triggers are stress, auditory triggers, fatigue, fasting, hormonal triggers, sleep, weather, visual triggers, olfactory triggers, and alcohol [9]. Dietary factors are less common, but their role in precipitating migraine episodes is of increasing interest. The classical and most frequently cited migraine dietary triggers are chocolate, cheese, citrus fruits, nuts, processed meats, monosodium glutamate, aspartame, red wine, and coffee [7,58,59,60]. It is believed that when these foods are avoided headaches improve [61]. However, in the case of exposure, attacks appear usually within 12–24 h after ingestion [3]. Kelman found that migraine patients with triggers have more family members with migraines, but also a longer lifelong duration of migraines, a higher frequency and duration of attacks, a better response to acute medications, more premonitory symptoms, more comorbidity, and more sleep difficulties than individuals without triggers [6]. There are several hypotheses about the causative connections between trigger factors and migraine initiation. The mechanism by which dietary triggers affect migraines may be connected with the release of serotonin and norepinephrine, vasoconstriction or vasodilatation, or by direct stimulation of the brainstem, cortical neuronal pathways, and trigeminal ganglia [62]. Trigger factors may diminish the migraine threshold, making it easier to initiate attacks. Alternatively, some migraine triggers may induce events that lead to a migraine attack. Some triggers may induce cortical spreading depression in the hyperexcitable migraine brain. Others may activate trigeminal nociceptors in the meninges or the neurons in the trigeminal nuclear complex. Headaches with trigger factors have greater severity or migraine features [63]. Many patients recognize multiple dietary triggers; thus, it is possible that most dietary precipitants in isolation are insufficient to trigger an attack [10]. Besides, it is not always easy to identify triggers. One study revealed that migraine patients show poor awareness of their headache triggers and hardly recognize a trigger unless asked specifically [64]. Hougaard et al. experimentally provoked 27 migraine patients, reporting that light or exercise would trigger their attacks, giving them different types of photo stimulation and exercise, or a combination of both. Only 3 (11%) individuals reported attacks following provocation, meaning that patients’ beliefs about triggers may be misleading [65]. Furthermore, many authors highlight the methodological difficulties of investigating the link between trigger factors and migraines [10]. According to Lipton et al., many different designs for studying trigger factors exist, such as surveys of beliefs, paper diary studies, electronic diary studies, case cross-over studies, repeated measurement retrospective cohort studies, and clinical trials [8]. Unfortunately, the majority of studies simply ask patients to retrospectively recall their usual headache triggers, which measures beliefs about triggers but not the link between exposure and headache occurrence. Using electronic diaries seems to be one of the best study designs, because the data are time-stamped so that the sequence of datum entry is known [8]. Besides, prospective diary studies better elucidate the causal association between potential triggers and migraine development, given that retrospective trigger reporting is subject to both recall and confirmation bias [66].

Many doctors, scientists, medical staff, and internet resources recommend avoiding triggers (including chocolate) to migraine patients [5,67,68]. Nevertheless, the common advice to identify and avoid triggers may be wrong. First of all, strict avoidance may lead to frustration, which in turn eliminates a potentially beneficial effect or even aggravates the situation. Besides, if a migraine is a disorder of the habituation of the CNS to sensory signals, the brain should be trained to habituate rather than avoid the triggers [51]. It is possible that avoiding triggers results in increased sensitivity to triggers. Moreover, studies have demonstrated that short exposure to a headache trigger results in increased sensitivity, and prolonged exposure results in decreased sensitivity (leading to desensitization). According to Martin et al., patients with migraines should cope with triggers rather than avoiding them [69]. Besides, there is no scientific evidence to recommend the so-called “migraine elimination diet” to patients.

## 2. Methods

This review includes all articles concerning the association between migraines and chocolate that have been published up to January 2020. The list of studies was obtained by searching clinical databases, including the MEDLINE, PubMed, Google Scholar, and the Cochrane library databases. Papers concerning any connection between chocolate/cocoa and headaches were identified through a literature search. The following terminology and keywords were applied: “chocolate”, “cocoa”, “cacao”, “migraine”, “headache”, “trigger factors”, “premonitory symptoms”, and “pain”. Each article obtained was then cross-referenced to identify relevant studies. Studies were eligible for inclusion if they were written in the English language. All types of articles, including observational, cross-sectional, and case-control studies, as well as clinical trials, were included and reviewed. The data were extracted from each article by two independent investigators.

## 3. Results and Discussion

Studies investigating the prevalence of chocolate as a trigger factor in patients with migraines are summarized in Table 1, and studies including provocative studies evaluating the effect of chocolate on migraine attacks are summarized in Table 2.

### 3.1. The Prevalence of Chocolate as a Migraine Trigger Factor

Twenty-five studies have evaluated the prevalence of chocolate as a migraine trigger. Among them, two studies failed to find any participant that reported chocolate as a trigger. In other studies, chocolate was found to be a migraine trigger in a small percentage of participants (ranging from 1.3 to 33). Nevertheless, it is worth noting that the majority of studies asked patients to retrospectively recall their usual headache triggers using a predetermined list of triggers, thus mostly assessing beliefs about triggers rather than facts. Only one study used an electronic diary (supposed to be one of the best trigger factor study designs) and found chocolate as a trigger in a very small percentage of migraineurs (<1.5%) [63]. This study also showed that headaches with triggers were significantly related to a greater pain intensity, headache-related disability, and the use of abortive treatment. Tai et al. found that chocolate and coffee significantly triggered migraines when compared to tension type headaches (TTHs) [61]. Besides, chocolate was the second most frequently implicated trigger factor among the migraine patients in the study [61]. Similarly, other studies have also confirmed the connection between chocolate and migraines when compared to TTH and have shown no association between TTH and chocolate intake [36,76,78,79]. A study evaluating TTH in migraine and non-migraine populations found that only migraine patients had episodes of tension-type headaches precipitated by alcohol, over-matured cheese, chocolate, and physical activity [79]. Neut et al. found that chocolate was a trigger in more migraine-with-aura individuals as compared to those with migraines without aura [74]. One study found no differences between episodic and chronic migraine patients in reporting triggers [34]. Taheri et al. examined the effect of the exclusion of certain frequently consumed dietary triggers in a population of children with headaches. Cocoa was identified as a trigger in 22% of children. After exclusion of the identified food triggers (1 to 3) for 3 months, 87% of patients achieved a complete resolution of headaches [71]. Rist et al. evaluated the association between the headache status and the low intake of foods commonly reported to trigger migraines. They discovered that those who experience non-migraine headaches were less likely to have a low intake of chocolate, which suggests that they consume more of those items than participants with no history of headaches. In addition they showed that migraine patients who experience auras were more likely to have a low intake of chocolate [73]. In a very interesting study, Wober et al. aimed to assess whether a certain factor precipitates headaches consistently or only occasionally and compared patients’ personal experiences to their theoretical knowledge about trigger factors. They revealed that the number of patients having heard or read that a certain factor might precipitate a headache was larger than the number of patients who actually experienced this factor as a trigger. The largest difference between the theoretical knowledge and personal experience was found for oral contraceptives (65.0% vs. 14.7%, *p* < 0.001) and chocolate (61.7% vs. 14.3%, *p* < 0.001) [76]. One study discovered that patients who had relatives with migraines were more likely to report the initiation of an attack by chocolate [81]. Dalton et al. recorded the food intake of 1883 women during the 24 h before a migraine attack, examining 2313 attacks. Chocolate was consumed prior to an attack by 33% of patients, and among them only 14% mentioned food when asked “what do you think caused this attack?” [37]. Another diary study found that migraines were more common on days with exposure to chocolate consumption, but only in 2.5% of patients [72]. Peris et al. found individual trigger profiles, with an average of four trigger factors linked to an increased risk of attack. They concluded that it is likely that a combination of triggers are associated with the occurrence of migraine attacks in individuals when a ‘threshold’ is reached [72]. One study revealed that having relatives with migraines was linked with chocolate being a trigger factor, which may suggest a genetic origin of response to triggers [81]. It was also shown that individuals who reported that alcohol, cheese, or citrus fruit provoked their headache were more likely to have migraines also triggered by chocolate, with the strongest correlation between sensitivity to alcohol and to these three food stuffs [81]. Dalton demonstrated that chocolate was a more prevalent migraine trigger in younger women, as compared to those over 50, and also in women who had a hysterectomy [37]. Besides, chocolate was reported as a trigger more frequently between 1–4 days into a menstrual cycle as compared with other days [37].

### 3.2. Double Blind Provocative Studies

Only three double blind studies have evaluated if chocolate can provoke migraine attacks. Two of them used carob as a placebo, while in the other the placebo consisted of synthetic fat. Marcus et al., in a study using chocolate or carob to induce headaches, found no difference between chocolate and the placebo in provoking headaches, regardless of personal beliefs about the possibility that chocolate provokes headaches. Before the study, a double-blind taste test of the chocolate and carob products was performed, in order to determine if it is possible to identify which sample contained the actual chocolate product. The results demonstrated that subjects could not accurately determine what they were eating, indicating that carob was an appropriate placebo for chocolate. Before the study, eleven subjects (17.5%) reported that chocolate was a trigger for their headaches. It is worth noting that all subjects initially followed a diet with the restriction of vasoactive amine-rich foods. The authors concluded that eating chocolate is not likely to be a trigger, but cited sweet food cravings as a part of prodrome phase of headaches [78]. Contrary to these results, Gib et al., in similar study, found that 41.7% of patients experienced migraine headaches after eating chocolate, while none developed headaches after eating a placebo [83]. However, the sample was small, and this difference did not reach a statistical significance. It is worth noting that the median time from eating chocolate to the onset of the attack was 22 h [83]. In the last study, Moffett et al. found no significant difference in the responses to chocolate or a placebo in a group of 25 migraine patients who reported chocolate as a headache trigger. In this study, 25 headaches were reported in 80 subject sessions, but only 13 of these occurred after eating chocolate alone [82].

## 4. Conclusions

A small proportion of migraine patients report chocolate as a trigger factor. However, it may be difficult to distinguish between migraine triggers and premonitory symptoms, as eating chocolate before attacks may be a result of food cravings. All provocative studies have failed to confirm that chocolate can trigger migraine attacks. Many possible mechanisms through which chocolate can influence migraines exist, and more are beneficial than unfavorable. Although there is a link between chocolate and migraines, a larger prospective study based on electronic diaries should be performed to assess the connection. Based on our review of the current literature, there is insufficient evidence that chocolate is a migraine trigger; thus, doctors should not make implicit recommendations to migraine patients to avoid it.

## Figures and Tables

**Table 1:** Overview of studies investigating the prevalence of chocolate as a trigger factor in migraineurs. Abbreviations: TTH–tension-type headache, MWA–migraine without aura, MA–migraine with aura, EM–episodic migraine, CM–chronic migraine, TF–trigger factor.

Author (Year)	Study Design	Study Design (Method of Identifying Trigger Factors)	Study Group: Type of Headache (Number of Participants)	Study Population Age (Years)	Chocolate/Cocoa Reported as a Trigger Factor (%)	Additional Information
Beh, S.C., 2019 [70]	Retrospective cross-sectional	Retrospective chart review	Vestibular migraine (*n* = 131)	No data	3.8	
Tai, M. S., 2018 [61]	Prospective cross-sectional	Comprehensive dietary check list	Migraine (*n* = 319) TTH (*n* = 365) MWA (*n* = 188) MA (*n* = 128), CM (*n* = 91)	Migraine 37.1 ± 14.3 TTH 46.5 ± 18.1	Migraine 11.6% TTH 3.8%	Chocolate was significantly associated with migraines compared to TTH.
Taheri, S., 2017 [71]	Prospective observational case series	Food diary	Migraine (*n* = 65) TTH (*n* = 50)	Range 10–15 Mean 10.5	22	87% of patients achieved complete resolution of headaches by the exclusion of 1–3 triggers
Park, J.W., 2016 [63]	Prospective cross-sectional	Smartphone headache diary application	Episodic Migraine (*n* = 62) MWA (*n* = 60) MA (*n* = 2)	Mean 37.7 ± 8.6	Cheese/ Chocolate 1.5	
Peris, F., 2016 [72]	Prospective cross-sectional	Detailed 90-day paper diary database from the PAMINA migraine study	Migraine (*n* = 326)	No data	2.5	
Constantinides, V., 2015 [36]	Prospective cross-sectional	Interview	Migraine (*n* = 21) MWA (*n* = 39) MA (*n* = 12) TTH (*n* = 12)	Migraine 41.4 ± 12.9 TTH 37.5 ± 15.5	Migraine 11.4 TTH 0	There was a tendency toward more frequent reports of chocolate as a trigger in migraine patients.
Rist, P., 2014 [73]	Cross-sectional study among participants in the Women’s Health Study	Semi-quantitative food frequency questionnaire	Non-migraine headache (*n* = 5573) Migraine (*n* = 7042) MWA (*n* = 2972) MA (*n* = 1974)	Mean 53.6Mean 53.6	Not applicable	Migraine patients with an aura were more likely to have a low intake of chocolate. Patients with non-migraine headaches were less likely to have a low intake of chocolate.
Mollaoglu, 2013 [7]	Prospective cross-sectional	Interview TF checklist	Migraine (*n* = 146) MWA (*n* = 73) MA (*n* = 53)	Mean 36.32	18.3	
Camboim Rockett, F., 2012 [35]	Cross-sectional study	Predetermined list of 22 dietary factors	Migraine (*n* = 123) MWA (*n* = 84) MA (*n* = 39)	Mean 43.2 ± 13.9	<20	
Neut, D., 2012 [74]	Retrospective	Predetermined list of TF	Migraine (*n* = 102) MWA (*n* = 71) MA (*n* = 22)	Mean 12 Range 7–16	11.8	
Finocchi, C. 2012 [3]	Prospective cross-sectional	No data	Migraine without aura (*n* = 100)	Mean 41.7 ± 14.2	20% of migraine attacks were triggered by food, among them 45% from chocolate	
Schürks, M., 2011 [75]	Cross-sectional study	Mailed migraine-specific questionnaire	Women’s Health Study (*n* = 1675)	No data	24.7	
Yadav, R., 2010 [40]	Prospective cross-sectional	Questionnaire	Migraine without aura (*n* = 182)	Mean 30.7 Range 14–58	None	None of the subjects reported chocolate as a trigger.
Andress-Rothrock, D., 2000 [34]	Prospective cross-sectional	Headache trigger checklist	Migraine (*n* = 200) EM (*n* = 56) CM (*n* = 144)	Mean 41.1 Range 16–75	3	
Chakravarty, A., 2009 [4]	Prospective and retrospective cross-sectional	Migraine trigger checklist	Migraine (*n* = 200) MWA (*n* = 197) MA (*n* = 3)	Range 7–15	1.5 retrospective study 0.3 prospective study	
Fukui, P., 2008 [5]	Prospective cross-sectional	Predetermined list of TGG	Migraine (*n* = 200)	Mean 37.7	20.5 (22.84% females, 10.53% males)	
Wöber, C., 2006 [76]	Cross-sectional study	Two predetermined TF checklists (patients’ personal experience and theoretical knowledge)	Migraine (*n* = 71) TTH (*n* = 49)	Range 18–65 Migraine 36.8 ± 11.4 TTH 39.5 ± 12.7	Theoretical knowledge 61.7 Personal experience 14.3	The difference between theoretical knowledge and personal experience was statistically significant and the largest for chocolate.
Takeschima, T., 2004 [39]	Door to door survey	Structured questionnaires	headache (*n* = 1628) migraine (*n* = 342) MWA (*n* = 301) MA (*n* = 41)	No data	None	
Bank, J., 2000 [77]	Population-based epidemiological survey	Self-administered headache questionnaire	Migraine (*n* = 62)	Mean Women 41 Men 43	1.4	
Marcus, D., 1997 [78]	Double-blind study		Headache (*n* = 63), 50% migraine, 37.5% TTH, 12.5 migraine + TTH	Mean 28.3	17.5	No significant difference of migraine attacks between chocolate and placebo.
Ulrich, 1996 [79]	A cross-sectional study	Mailed questionnaire	Migraine (*n* = 484) MWA (*n* = 342) MA (*n* = 163)	No data	1.7	Only migraineurs experienced chocolate as a precipitant of tension-type headaches
Van Den Bergh, 1987 [80]	Retrospective	Unstructured recall/free self-report	Migraine (*n* = 217)	Mean 40	22.5	
Peatfield, R., 1984 [81]	Retrospective cross sectional	Interview	Migraine (*n* = 490)	No data	19	
Dalton, 1975 [37]	Prospective cross- sectional	Self-administered postal questionnaire	Migraine in women (*n* = 1883)	No data	33	
Moffet, A.M., 1974 [82]	Retrospective study	Questionnaire	Migraine (*n* = 332)	No data	26.5	

**Table 2:** Overview of provocative studies evaluating the effect of chocolate on migraine attacks. Abbreviations: TTH–tension type headache.

Author (Year)	Study Design	Placebo	Chocolate/Placebo Amount	Study Group: Type of Headache (Number of Participants)	Study Population Age (Years)	Chocolate/Cocoa Reported as a Trigger Factor (%) before the Study	Conclusion
Marcus, D., 1997 [78]	Double-blind study	Carob	60 g	Headache (*n* = 63), 50% migraine, 37.5% TTH, 12.5 migraine + TTH	28,3	17.5	No significant difference of migraine attacks between chocolate and placebo.
Gibb, C., 1991 [83]	Double-blind, placebo controlled trial	Carob powder, coberine (non-cocoa vegetable fat)	40 g	Migraine (*n* = 20)	Chocolate 37 Placebo 42	100	41.7% developed headaches after chocolate ingestion, none after placebo.
Moffet, A.M., 1974 [82]	Double-blind, placebo controlled trial	Synthetic fat made from non-cocoa containing vegetable oils with added sugar, coloring, and flavoring	44 g	Migraine (*n* = 25)	Mean 49 Range 22–62	100	No significant difference of migraine attacks between chocolate and placebo.
